# Tick-Borne Flavivirus Inhibits Sphingomyelinase (*Is*SMase), a Venomous Spider Ortholog to Increase Sphingomyelin Lipid Levels for Its Survival in *Ixodes scapularis* Ticks

**DOI:** 10.3389/fcimb.2020.00244

**Published:** 2020-06-12

**Authors:** Pravesh Regmi, Supreet Khanal, Girish Neelakanta, Hameeda Sultana

**Affiliations:** ^1^Department of Biological Sciences, Old Dominion University, Norfolk, VA, United States; ^2^Division of Infectious Diseases and International Health, Department of Medicine, University of Virginia School of Medicine, Charlottesville, VA, United States

**Keywords:** ticks, *Is*SMase, Langat Virus (LGTV), GW4869 inhibitor, sphingomyelinase, sphingomyelin, exosomes

## Abstract

Our previous study showed that cells from medically important arthropods, such as ticks, secrete extracellular vesicles (EVs) including exosomes that mediate transmission of flavivirus RNA and proteins to the human cells. Understanding the molecular determinants and mechanism(s) of arthropod-borne flavivirus transmission via exosome biogenesis is very important. In this current study, we showed that in the presence of tick-borne Langat Virus (LGTV; a member of tick-borne encephalitis virus complex), the expression of arthropod *Is*SMase, a sphingomyelinase D (SMase D) that catalyzes the hydrolytic cleavage of substrates like sphingomyelin (SM) lipids, was significantly reduced in both *Ixodes scapularis* ticks (*in vivo*) and in tick cells (*in vitro*). The *Is*SMase reduced levels correlated with down-regulation of its activity upon LGTV replication in tick cells. Our data show that LGTV-mediated suppression of *Is*SMase allowed accumulation of SM lipid levels that supported membrane-associated viral replication and exosome biogenesis. Inhibition of viral loads and SM lipid built up upon GW4869 inhibitor treatment reversed the *Is*SMase levels and restored its activity. Our results suggest an important role for this spider venomous ortholog *Is*SMase in regulating viral replication associated with membrane-bound SM lipids in ticks. In summary, our study not only suggests a novel role for arthropod *Is*SMase in tick-LGTV interactions but also provides new insights into its important function in vector defense mechanism(s) against tick-borne virus infection and in anti-viral pathway(s).

## Introduction

Vector-borne diseases that account for high morbidity and mortality throughout the world have been of major concern (Powell, 2019; Qurollo, 2019; Scalway et al., 2019; Shaw and Catteruccia, 2019; Spence Beaulieu, 2019; Wilke et al., 2019; Wilson et al., 2019). The medically important *Ixodes scapularis* tick transmits a variety of pathogens that cause severe diseases in humans and animals (Labuda et al., 1996; Labuda and Randolph, 1999; Nuttall et al., 2000; Nuttall and Labuda, 2003, 2004; Piesman and Eisen, 2008; Neelakanta and Sultana, 2015; de la Fuente, 2018; Kim, 2019). Some of the tick-borne pathogens of human health importance are the newly emerging Powassan virus (POWV), tick-borne encephalitis virus (TBEV), Lyme disease agent *Borrelia burgdorferi*, rickettsial pathogens (such as *Rickettsia rickettsii* and *Anaplasma phagocytophilum*), and *Francisella tularensis*, a bacterium causing tularemia (Randolph et al., 1996; Sexton and Kirkland, 1998; Nuttall et al., 2000; Piesman and Eisen, 2008; Sultana et al., 2010; Valarcher et al., 2015; Vora et al., 2017; Zhou et al., 2018; Sekeyova et al., 2019; Zellner and Huntley, 2019). Ticks have evolved a myriad of strategies that allow them to get a blood meal through feeding on a vertebrate host for several days. The presence of pharmacological agents in tick's saliva modulates pain, itch, blood clotting, wound healing, immune responses, and inflammation that allows longer feeding periods and sufficient time for the transmission of pathogens to a variety of vertebrate host (Nuttall et al., 2000; Nuttall and Labuda, 2003, 2004). A study has identified a novel sphingomyelinase-like enzyme (*Is*SMase) in *I. scapularis* tick saliva that modulates the adaptive immune response by inclining the host CD4+ T-cells to result in a shift from a neutralizing Th1 cytokine response toward a Th2-induced cytokine profile response (Alarcon-Chaidez et al., 2009). This Mg^+2^-dependent, neutral form of *Is*SMase directly (independent of its enzymatic activity) programmed the CD4+ T cells in order to express interleukin 4 (IL-4), which is a hallmark of Th2 effects (Alarcon-Chaidez et al., 2009). *Is*SMase showed high homology to the *Loxosceles* venomous spider's sphingomyelinase D (SMase D) protein (Alarcon-Chaidez et al., 2009). SMase D is also known as phospholipase D (PLD1) or sphingomyelin (SM) phosphodiesterase D that catalyzes the hydrolytic cleavage of substrates like SM or lysophospholipid, such as lysophosphotidylcholine resulting in the formation of choline and ceramide 1-phosphate or choline and lysophosphatidic acid (LPA), respectively (Truett, 1993; Zager et al., 2000; Binford et al., 2009; Zobel-Thropp et al., 2010; Dias-Lopes et al., 2013). SM (with generic name *N*-acyl-sphingosine-l-phosphorylcholine) is a major structural component of the plasma membrane in all eukaryotic cells that performs biological functions together with other phospholipids, glycolipids, cholesterol, and membrane-integrated proteins (Truett, 1993; Zager et al., 2000; Zobel-Thropp et al., 2010; Schneider-Schaulies and Schneider-Schaulies, 2015). By-products of SM such as ceramide, sphingosine, and sphingosine-l-phosphate are essential cellular effectors playing roles in apoptosis, cell development, and survival (Kolesnick and Kronke, 1998; Chmura et al., 2000; Bikman and Summers, 2011; Vijayan and Hahm, 2014; Schneider-Schaulies and Schneider-Schaulies, 2015; Bezgovsek et al., 2018; Soudani et al., 2019). It has been shown that SMase D, the main toxin in the spider venom, causes dermonecrotic lesions on mammalian skin, which are characteristic of envenomation by the *Loxosceles* spiders (Zobel-Thropp et al., 2010). *Loxosceles intermedia* Class 2 venom SMase D has shown to increase the production and secretion of matrix metalloproteinases (MMPs) 2 and 9, whereas the more potent Class 1 SMase D from *L. laeta* induced MMP7 in addition to MMP2 and 9, thereby resulting in keratinocyte death (Paixao-Cavalcante et al., 2007; Correa et al., 2016). It is interesting to note that spider's SMase D is responsible for local skin necrosis in general, and in occasional cases, it also results in severe systemic manifestations, such as acute kidney failure and death (Paixao-Cavalcante et al., 2007; Zobel-Thropp et al., 2010; Correa et al., 2016).

A bioinformatics analysis identified SMase D or proteins with SMase D activity in bacteria (*Corynebacteria* and *Arcanobacterium*), fungi (*Aspergillus* and *Coccidioides*), mites, spiders, and tick saliva (Binford et al., 2009; Zobel-Thropp et al., 2010; Dias-Lopes et al., 2013; Lopes et al., 2013; Pedroso et al., 2015). A common C-terminal similar motif (SMD-tail) at the end of spider's SMase D supported the identification of the broadly conserved glycerophosphoryl-diester phosphodiesterase (GDPD) family in different organisms (Binford et al., 2005). Phylogenetic analysis of spider's SMase D showed a possible convergent evolution that is independent from GDPD ancestor such as fungi and arthropods (Zobel-Thropp et al., 2010; Dias-Lopes et al., 2013). Tick *Is*SMase recombinant protein intradermal injections (at 5 μg) showed no dermonecrotic activity or any detectable cutaneous changes or inflammation (Alarcon-Chaidez et al., 2009). However, in the presence of salivary contents, *Is*SMase may contribute to the feeding lesions at the tick bite site (Alarcon-Chaidez et al., 2009). In addition to *I. scapularis* ticks, SMase D has been identified in other genera of ticks such as *Amblyomma* and *Rhiphicephalus*, where this toxin is shown to be involved in animal ear inflammation and possible necrosis (Binder, 1989; Truett, 1993; Binford et al., 2005, 2009). Our previous study showed that both positive and negative strands of LGTV RNA and viral envelope (E), non-structural 1 (NS1) protein, and perhaps poly-protein were contained inside exosomes derived from ISE6 tick cells (Zhou et al., 2018). These tick cell-derived exosomes were enriched with exosomal marker HSP70 and infection with LGTV induced exosome production and release as a means of induced viral RNA and protein dissemination (Zhou et al., 2018). This study indicates a role for sphingomyelinases in affecting the exosome's biogenesis, viral replication, and dissemination. In this current study, we not only report a novel role for *Is*SMase in tick-LGTV interactions but also delineate its important function in tick anti-viral pathway(s).

## Materials and Methods

### Bioinformatics and Prediction Analysis

The amino acid sequences of *Is*SMase were used from GenBank and analyzed at Prosite website (http://prosite.expasy.org). The deduced *Is*SMase amino acid sequence is aligned (with other orthologs) using ClustalW program in DNASTAR Lasergene. Matching residues are shaded in black color for easy identification. Annotation/prediction analysis performed in CLC Genomics Workbench 20.0 for *Is*SMase protein sequence is shown. Prosite from Expasy (https://prosite.expasy.org/) and NCBI CDD search (https://www.ncbi.nlm.nih.gov/Structure/cdd/wrpsb.cgi) was used for the prediction of glycosylation, myristoylation, protein kinase C (PKC) phosphorylation, casein kinase II phosphorylation, tyrosine phosphorylation, and cAMP-dependent protein kinase phosphorylation sites in *Is*SMase.

### GenBank Accession Numbers

The GenBank accession numbers for the sphingomyelinase sequences used in this study are as follows: *I. scapularis* sphingomyelinase-like enzyme (*Is*SMase; accession number ABD73957), *Rhipicephalus pulchellus* (SM phosphodiesterase; accession number JAA56531), *Amblyomma maculatum* (hypothetical protein; accession number AEO33547), and spider orthologs such as *Scicarius patagonicus* (SMase D; accession number C0JB69), *Hemiscorpius lepturus* (venom toxin; accession number API81381) and *L. similis* (loxtox protein; accession number ANY30961).

### Ticks, Synchronous Infections, and Tick Feeding on Mice

Unfed nymphal *I. scapularis* ticks were obtained from BEI resources (ATCC)/Centers for Disease Prevention and Control (CDC) and were maintained in our laboratory. Ticks were kept at room temperature with approximately 98% relative humidity under a photoperiod of 14 h of light and 10 h of darkness. For the expression of *Is*SMase, we used three different developmental stages of ticks: larvae, nymphs, and adults (male or female) obtained from BEI Resources/CDC. For synchronous infection, we followed published protocols (Mitzel et al., 2007; Taank et al., 2018). Unfed nymphs were collected in sterile 1.5-ml Eppendorf tubes. Out of 48 nymphs (used in total), 24 (12 in each tube) were maintained as uninfected controls and 24 (12 in each tube) were synchronously infected with LGTV (LGT-TP21 strain). Briefly, nymphs were infected by immersion into 0.5 ml of complete Dulbecco's modified eagle's medium (DMEM) containing 1 × 10^7^ plaque-forming units (pfu)/ml of LGTV. For the uninfected control group, nymphs were immersed into DMEM without virus. The tubes were incubated for 45 min at 34°C (with tubes being vortexed every 10 min to redistribute ticks in the media). Tubes were then chilled on ice (for 2 min) and centrifuged at 200 × g for 30 s. Nymphs were washed twice with cold 1 × PBS by centrifugation. After washing, ticks were dried with Whatman paper and transferred into sterile collection tubes with holed caps covered with nylon mesh cloth. LGTV-infected and uninfected nymphs were kept in separate tubes with proper labels in an environmental chamber maintained at room temperature and a relative humidity of 98% for 17 days. Ticks generated by this synchronous method were used as LGTV-infected unfed ticks. LGTV-infected ticks were partially fed (for 24 h during feeding- DF) on wild-type C57BL/6 mice (purchased from Charles River Laboratories) for 24 h and ticks were pulled with forceps. Uninfected ticks fed on naïve C57BL/6 mice were used as controls. All experiments were conducted in strict accordance with the recommendations in the *Guide for the Care and Use of Laboratory Animals* of the NIH, USA, and using approved protocol from the Institutional Animal Care and Use Committee (IACUC; protocol #18-011).

### *In vitro* Tick Cell Culture, Infections, and Exosome Isolation From Cell Culture Supernatants

We used *I. scapularis* ISE6 tick cell line that is grown and maintained as described in our previous study (Zhou et al., 2018). Wild-type LGTV (LGT-TP21) strain used in this study is maintained in Vero cells as laboratory virus stocks. Briefly, 5 × 10^5^ cells were seeded in 12-well plates and infected with LGTV [with a multiplication of infection (MOI) of 1, for time points 24, 48, and 72 h post-infection (p.i.), or with various MOI doses of 1–3; for dose response collections at 72 h p.i.]. Exosomes were isolated from tick cell culture supernatants by a differential ultracentrifugation method (Vora et al., 2018; Zhou et al., 2018, 2019). Details for these procedures are also schematically shown in our previous study (Zhou et al., 2018). To isolate exosomes, we used concentrated cell culture supernatants. Purified exosome preparations were stored at −80°C and used for further analysis.

### RNA Extraction, cDNA Synthesis, and QRT-PCR Analysis

Total RNA was extracted from both uninfected and LGTV-infected nymphal ticks (unfed or 24 h partially fed) or ISE6 tick cells or exosomes derived from tick cells, using Aurum Total RNA Mini kit (Bio-Rad) and following the manufacturer's instruction. RNA samples generated (in our previous studies) from *B. burgdorferi*- or *A. phagocytophilum*-infected unfed nymphs (Khanal et al., 2018; Taank et al., 2018) were used in this study. RNA (1 μg) was converted into cDNA using iScript cDNA synthesis kit (Bio-Rad). The generated cDNA was used as a template to amplify and determine the viral burdens and *Is*SMase levels by performing quantitative real-time PCR (QRT-PCR) using the iQ-SYBR Green Supermix kit (Bio-Rad) and by following the manufacturer's instructions. We used a Bio-Rad CFX96 QPCR machine to perform the QRT-PCR reactions. Published forward and reverse primers were used to detect LGTV-RNA (Zhou et al., 2018). *Is*SMase transcripts were detected using primer pairs 5′ CGCCGCTGGAGTAGACATC 3′ and 5′ GACCCACATCGAATCCCACA 3′. The *hsp70* transcripts were amplified using published primers (Vora et al., 2017). Tick beta-actin levels were quantified using published primers (Taank et al., 2018; Zhou et al., 2018) and were used to normalize the levels of other transcripts in all analysis. In addition to normalizing with tick actin, LGTV prM-E levels were also normalized to the total RNA levels that are shown on the *Y*-axis. Equal volume of cDNA was used in parallel for LGTV prM-E, beta-actin, *hsp70*, and *issmase* primers. Standard curves were prepared from each gene standard using 10-fold serial dilutions starting from standard 1 as 1 ng to standard 6 as 0.000001 ng of known quantities of *actin*. Untreated samples served as internal controls.

### GW4869 Inhibitor Studies

We used GW4869, a cell-permeable, selective inhibitor for neutral sphingomyelinase (N-SMase) (obtained from Santa Cruz Biotechnologies, Inc). Tick cells showed no cytotoxicity with 1 μM of GW4869 inhibitor treatment and hence we considered this dose in both experiments. DMSO was used as a vehicle control. A similar volume of DMSO that is equal to 1 μM GW4869 was used in our experiments. Tick cells were plated and incubated overnight and then treated with 1 μM GW4869 inhibitor for 4 h, followed by infection with LGTV for 72 h p.i. Before infection, we did not wash cells to remove the inhibitor.

### Sphingomyelinase and SM Quantification Assays

We used colorimetric sphingomyelinase or SM quantification assay kits from Sigma-Aldrich (USA) and followed the manufacturer's instructions. Sphingomyelinase assay was performed as described in our recent study (Zhou et al., 2019). Briefly, we plated 5 × 10^5^ tick cells, and after overnight incubation, cells were infected with LGTV (MOI 1) for either 24 or 72 h (p.i.) for both assays. Cell lysates were resuspended in 1 × PBS and processed for sphingomyelinase activity or SM lipid levels, immediately. For each time point and reaction well, 50 μl of samples (uninfected or LGTV-infected) was used as six replicates. Zero blank sphingomyelinase/SM standards were considered as background values, respectively, in each assay. Samples from sphingomyelinase or SM assays were measured at 655 or 570 nm absorbance, respectively. Using the standard values, curves were plotted and the amount of enzyme or lipid present in the samples was determined from the standard curve.

### Statistics

Statistical differences observed in data sets were analyzed using GraphPad Prism6 software and Microsoft Excel. The non-paired, two-tailed Student's *t*-test was performed (for data to compare two means) for the entire analysis. Each experiment was performed for three independent times and 5–10 replicates were considered in each experiment. Error bars represent mean (±SD) values, and *P*-values of < 0.05 were considered significant in all analyses.

## Results

### Detailed Bioinformatics Analysis Revealed *I. scapularis* Sphingomyelinase (*Is*SMase) as a Potential Venomous Protein Ortholog From Spiders

**Figure 1:**
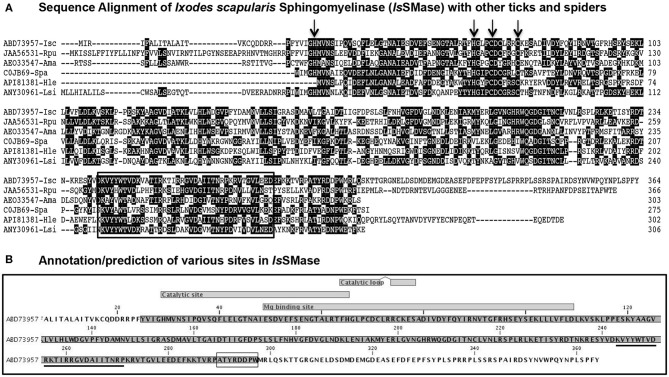
Sequence alignments and prediction analysis of *Is*SMase with tick and spider orthologs. **(A)** The deduced *I. scapularis* (*Isc*) SMase (*Is*SMase) amino acid sequence alignment (with other orthologs) using ClustalW program in DNASTAR Lasergene is shown. Matching residues are shaded in black color. GenBank accession numbers for *R. pulchellus* (*Rpu*) sphingomyelin phosphodiesterase, *A. maculatum* (*Ama*) hypothetical protein, *S. patagonicus* (*Spa*) sphingomyelinase D, *H. lepturus* (*Hle*) venom toxin, and *L. similis* (*Lsi*) loxtox protein sequences are shown. GenBank accession numbers for *Isc, Rpu*, and *Ama* are provided. Total length of the amino acid sequence is provided at the right end of each sequence. **(B)** Annotation/prediction analysis performed in CLC Genomics Workbench 20.0 for *Is*SMase protein sequence is shown. The catalytic site and magnesium binding sites and their overlap site are shown. The underlined sequence indicates the glycero-phosphodiester phosphodiesterase-like motif. The SMaseD consensus motif is shown as a boxed amino acid sequence.

In this study, we characterized *Is*SMase in detail. Expression of *issmase* gene transcripts was noted in uninfected tick cells, unfed nymphs, and 24 h partially fed nymphs (Supplementary Figure 1). We performed bioinformatics, comparative, and prediction analysis on *Is*SMase (accession number ABD73957) and compared its sequence with that of its orthologs from other ticks such as *R. pulchellus* (SM phosphodiesterase; JAA56531), *A. maculatum* (hypothetical protein; AEO33547), and spider orthologs such as *S. patagonicus* (SMase D; C0JB69), *H. lepturus* (venom toxin; API81381), and *L. similis* (loxtox protein; ANY30961). ClustalW alignment of *Is*SMase with *R. pulchellus* or *A. maculatum* ticks showed less degree of conservation (Figure 1A), whereas *Is*SMase sequence comparison with *S. patagonicus, H. lepturus*, and *L. similis* spider orthologs revealed high conservation in the amino acid sequence (Figure 1A). Comparative sequence analysis of *Is*SMase protein with tick or spider orthologs found the conserved motif/domain glycerophosphoryl diester phosphodiesterases (GDPD-like SMase D-PLD) from the SMase D family (Figure 1A). This conserved motif/domain sequence is highlighted with a black box and the predicted leader peptide for *Is*SMase is underlined (Figures 1A,B). Domain analysis revealed that *Is*SMase shared catalytic sites with other SMase D orthologs. The residues (H34, H70, C76, and C80; highlighted with arrows) important for catalytic activity were conserved among all the analyzed SMases (Figure 1A). The catalytic site lies between residues 29 and 65 with two catalytic loops of residues 64–71 and 74–78 (Figure 1B). The magnesium binding site that overlapped with the catalytic site lies between residues 49/51 and 109 (Figure 1B).

**Figure 2:**
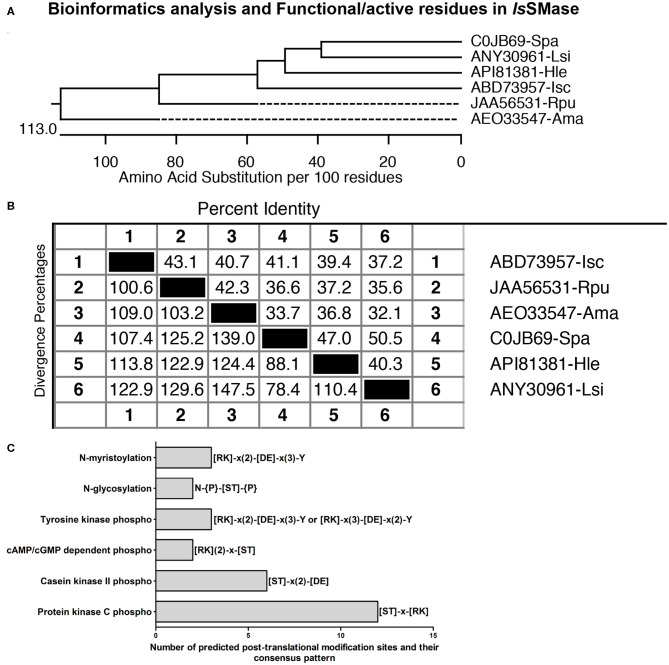
Comparative bioinformatics analysis of *Is*SMase with other orthologs. **(A)** Phylogenetic analysis comparing *Is*SMase and other orthologs was performed in DNASTAR by ClustalW slow/accurate alignment method using Gonnet as default value for protein weight matrix. Scale at the bottom denotes amino acid substitutions per 100 amino acid residues. **(B)** Percent identity (horizontally above black boxed diagonal) and divergence (vertically below black boxed diagonal) of *Is*SMase nucleotide sequence in comparison to *R. pulchellus* (*Rpu*) sphingomyelin phosphodiesterase, *A. maculatum* (*Ama*) hypothetical protein, *S. patagonicus* (*Spa*) sphingomyelinase D, *H. lepturus* (*Hle*) venom toxin, and *L. similis* (*Lsi*) loxtox protein sequence is shown. **(C)** Protein feature prediction analysis showing *Is*SMase protein modification sites and relevant amino acids as functionally active residues in those modifications. *Is*SMase contains 12 protein kinase C (PKC) phospho-sites, 6 casein kinase II phospho-sites, 2 cAMP/cGMP-dependent phospho-sites, 3 tyrosine kinase phospho-sites, 2 N-glycosylation sites, and 3 N-myristoylation sites.

Furthermore, the phylogenetic analysis showed that *Is*SMase is closely related to spider orthologs and forms a clade that clusters with the venomous spider ortholog SMase D (Figure 2A). However, both *R. pulchellus* and *A. maculatum* tick orthologs formed separate sub-clades, suggesting differences in SMases from these ticks (Figure 2A). Tick SMase D orthologs from *R. pulchellus* and *A. maculatum* also showed high degree of divergence to *Is*SMase and the spider orthologs (Figure 2B). *Is*SMase amino acid sequence comparison revealed 43.1 or 40.7% identity to *R. pulchellus* and *A. maculatum* ticks, and it showed 41.1, 39.4, and 37.2% identity with *S. patagonicus, H. lepturus*, and *L. similis* spider orthologs, respectively (Figure 2B). In addition, protein feature prediction analysis, performed as previously described (Turck et al., 2019), revealed the presence of 12 PKC phospho-sites (with 4 serine and 8 threonine residues), 6 casein kinase II phospho-sites (with 5 serine and 1 threonine residue), 2 cAMP/cGMP-dependent phospho-sites (with 1 each of serine and threonine residues), 3 tyrosine kinase phospho-sites (with 3 tyrosine residues), 2 N-glycosylation sites (with 2 N-linked GlcNAc; asparagine residues), and 3 N-myristoylation sites (with 3 glycine residues), respectively (Figure 2C). Presence of several protein modification sites suggests *Is*SMase to be a highly functional enzyme in ticks.

### *Is*SMase Is Not Developmentally Regulated in *I. scapularis* Ticks

**Figure 3:**
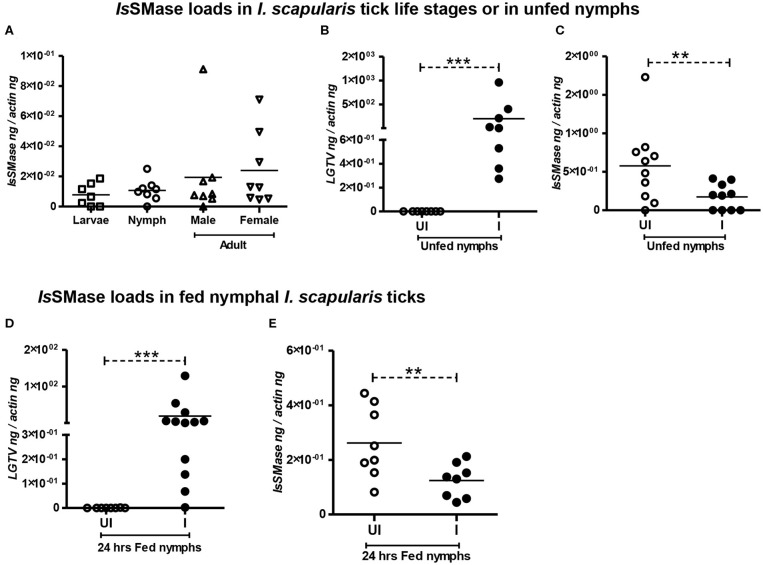
*Is*SMase expression is reduced upon LGTV infection in ticks. QRT-PCR analysis showing *Is*SMase gene expression levels in different developmental/life stages (larvae, nymphs, adult male, and female) of ticks **(A)**. LGTV viral loads **(B)** or *Is*SMase transcript loads **(C)** in unfed nymphs is shown. QRT-PCR showing LGTV viral loads **(D)** or *Is*SMase transcript loads **(E)** in partially fed (24 h) nymphs. UI indicates uninfected or I denotes LGTV-infected nymphs. Each square, circle, triangle, or inverted triangle indicates one tick. Open circles represent uninfected (UI) group, whereas closed circles denote LGTV-infected (I) group. LGTV loads or *Is*SMase mRNA levels were normalized to tick beta-actin levels. *P*-value determined by Student's two-tailed *t*-test is shown. The asterisk indicates significance, and ** or *** denotes a *P*-value of less than 0.01 or 0.001, respectively.

We analyzed the expression of *Is*SMase in three different stages of ticks: larvae, nymphs, and adults (male or female) to understand the importance of this molecule in tick life cycle and developmental stages. QRT-PCR analysis showed that *Is*SMase is expressed in all three developmental stages of ticks. *Is*SMase mRNA levels appeared to be higher in adult male and female ticks and low level of expression was noted in larval and nymphal ticks (Figure 3A). However, the levels of *Is*SMase in the three developmental stages of ticks showed no significant (*P* > 0.05) differences in its expression (Figure 3A). These data suggest that *Is*SMase expression is not influenced by developmental changes in ticks.

### *Is*SMase Expression Is Reduced Upon LGTV Infection in Both Unfed/Fed Ticks and in Tick Cells

Our previous study (Zhou et al., 2018) suggests analyzing the importance of *Is*SMase in ticks. Combing of *I. scapularis* genome revealed the presence of several sphingomyelinase-like enzymes of both acidic and basic types; however, we addressed the spider venomous SMase D ortholog *Is*SMase that was previously identified in ticks (Alarcon-Chaidez et al., 2009). QRT-PCR analysis revealed detection of LGTV loads in unfed nymphs that were generated by synchronous viral infection (see section Materials and Methods) (Figure 3B). Viral loads were highly detectable in all tested ticks, but the *Is*SMase transcript levels were significantly (*P* < 0.05) reduced in LGTV-infected unfed ticks (Figure 3C) in comparison to the uninfected controls. Next, we generated synchronously infected LGTV *I. scapularis* ticks and partially fed them on uninfected mice. Ticks were collected at 24 h during feeding. As expected, we found significantly (*P* < 0.05) higher viral burden determined by LGTV-prM-E transcript levels (Figure 3D). Similar to the observation noted with unfed nymphs, LGTV-infected ticks collected during 24 h of partial feeding also showed significantly (*P* < 0.05) reduced *Is*SMase transcript levels in comparison to the levels noted in uninfected controls (Figure 3E).

**Figure 4:**
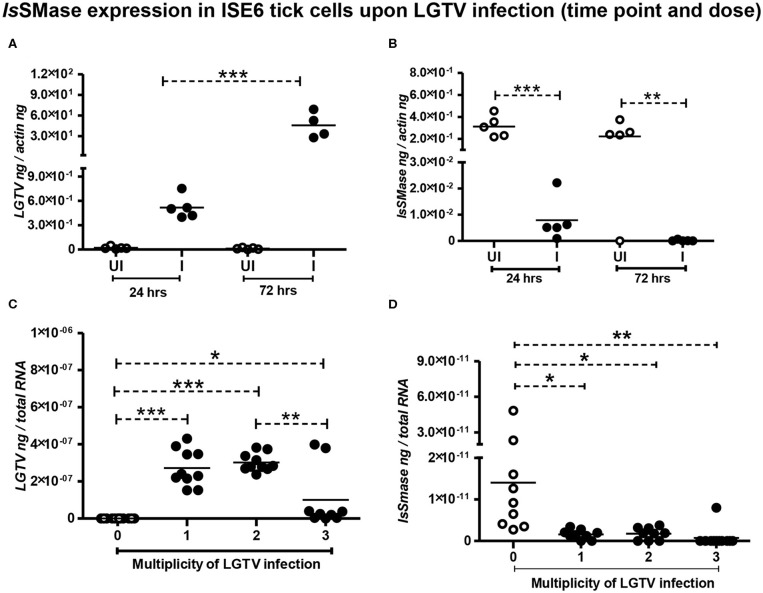
Reduced *Is*SMase expression upon LGTV infection is time and dose dependent. QRT-PCR analysis showing LGTV viral loads **(A,C)** or *Is*SMase gene expression levels **(B,D)** at different time points **(A,B)**, or doses **(C,D)** in tick cells. LGTV loads **(A)** and *Is*SMase transcript levels **(B)** were determined at two different time points of 24 and 72 h p.i. **(C,D)**, numbers (1, 2, 3) indicate multiplication of infection (MOI) corresponding to LGTV loads **(C)** or levels of *Is*SMase transcripts **(D)** in uninfected (0) or LGTV-infected (1, 2, 3 MOI) tick cells. Each circle indicates sample generated from one culture well analyzed as 5–10 replicates. Open circles represent the uninfected (UI or 0) group, whereas closed circles denote the LGTV-infected (1, 2, and 3 in dose or I in time points) group. LGTV loads or *Is*SMase mRNA levels were normalized to tick beta-actin levels. *P*-value determined by Student's two-tailed *t*-test is shown. The asterisk indicates significance, and *, **, or *** denotes a *P*-value of less than 0.05, 0.01, and 0.001, respectively.

We have previously shown that LGTV readily infected *I. scapularis* ISE6 tick cells, with increased viremia at 72 h p.i. (Zhou et al., 2018). Next, we determined the *Is*SMase levels in ISE6 tick cells. We first performed a time response of LGTV infection (with MOI 1) in tick cells by considering one early (24 h p.i.) and one late time point (72 h p.i.). QRT-PCR analysis showed significant (*P* < 0.05) induction in LGTV infection over the time course of 24 and 72 h p.i. (Figure 4A); however, in the same samples, we found that *Is*SMase transcript levels were significantly (*P* > 0.05) reduced at both tested time points in comparison to the respective uninfected controls (Figure 4B). In addition, we performed a dose-response experiment by infecting tick cells with various doses of LGTV (MOIs of 1, 2, and 3). We found that tick cells were tolerant for MOI 1 and 2; however, 25–30% of tick cells were susceptible to LGTV infection at MOI 3. QRT-PCR analysis revealed no significant differences in viral loads between tick cells that were infected with MOI 1 and 2 (Figure 4C). However, significant (*P* < 0.05) difference in viral loads was evident between tick cells infected with MOI 2 and 3 (Figure 4C). We found that *Is*SMase transcript levels were significantly (*P* < 0.05) lower at all tested MOIs in comparison to the uninfected control (Figure 4D). Reproducible to our previous study, we found that LGTV loads were abundantly present in tick cell-derived exosomes (Supplementary Figure 2A). We noticed the exosomal marker HSP70 transcript levels; however, we did not detect *Is*SMase transcript levels in tick cell-derived exosomes (Supplementary Figures 2B,C). Overall, these data suggest that LGTV infection reduces the levels of *Is*SMase in unfed nymphs, in partially fed nymphs (collected at 24 h during feeding), and in tick cells (in a time- and dose-dependent response).

### GW4869 Inhibitor Treatment Reduced LGTV Loads and Restored the *Is*SMase Transcript Levels

**Figure 5:**
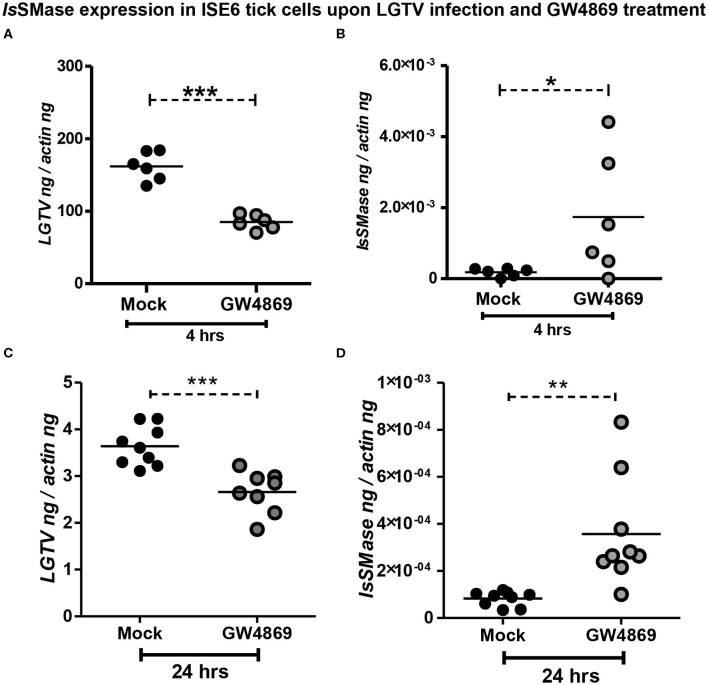
GW4869 treatment restored the *Is*SMase expression by inhibiting LGTV loads in tick cells. **(A)** QRT-PCR analysis showing LGTV loads **(A,C)** or *Is*SMase mRNA levels **(B,D)** upon pre-treatment of ISE6 tick cells with GW4869 (1 μM) for either 4 h **(A,B)** or 24 h **(C,D)** followed by LGTV infection (for 72 h p.i.). Both mock and GW4869 inhibitor-treated groups were infected with 1 MOI of LGTV. Mock represents the group treated with vehicle DMSO. Each circle indicates sample generated from one culture well and analyzed as 6–10 replicates. Black circles represent the LGTV-infected mock group, whereas gray circles denote the LGTV-infected GW4869 inhibitor-treated group. LGTV loads or *Is*SMase mRNA levels were normalized to tick beta-actin levels. *P*-value determined by Student's two-tailed *t*-test is shown. The asterisk indicates significance, and *, **, or *** denotes a *P*-value of less than 0.05, 0.01, and 0.001, respectively.

Dihydrochloride hydrate (GW4869) is a cell-permeable but selective inhibitor for neutral sphingomyelinase(s) that affects the exosome production and release. Our previous study showed that GW4869 inhibitor treatment reduced LGTV loads in exosomes and also inhibited the transmission of LGTV RNA and proteins via infectious exosomes to both tick (5 μM) and vertebrate (1–20 μM) host cells (Zhou et al., 2018). We determined whether GW4869 (1 μM) treatment for either 4 or 24 h also reduced the LGTV loads in tick cells. QRT-PCR analysis revealed that LGTV loads were significantly (*P* < 0.05) reduced at 4 h of GW4869 treatment (Figure 5A). The reduction in LGTV replication/loads correlated with significantly (*P* < 0.05) increased expression of *Is*SMase in these tick cells (Figure 5B). In addition, higher incubation times (for 24 h) of GW4869 treatment showed significant (*P* < 0.05) reduction of LGTV loads (Figure 5C) that significantly (*P* < 0.05) increased expression of *Is*SMase in these tick cells (Figure 5D). These data show a direct association of LGTV replication/loads in the suppression of the *Is*SMase levels in tick cells that are restored with GW4869 treatment.

### LGTV Reduced *Is*SMase Activity to Induce SM Lipid Accumulation

**Figure 6:**
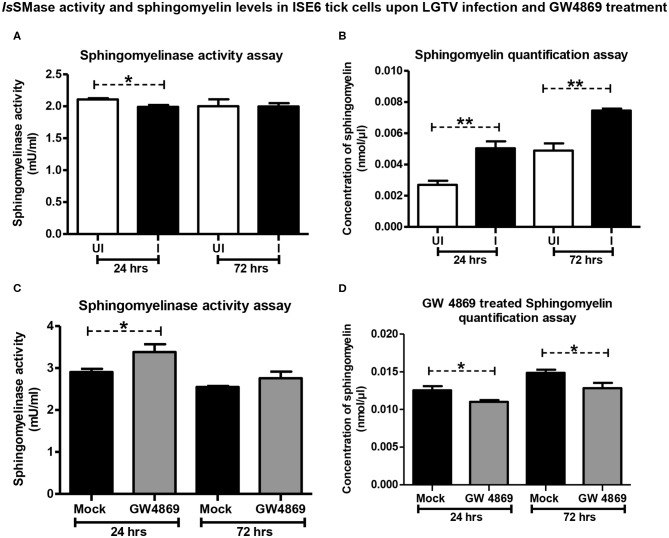
LGTV infection reduced *Is*SMase activity resulting in induced levels of sphingomyelin lipids but GW4869 treatment restored the infection-mediated effects. **(A)** Measurement of sphingomyelinase activity in uninfected or LGTV-infected tick cells **(A)** or mock or GW4869 (1 μM)-treated, LGTV-infected tick cells **(C)** at 24 and 72 h p.i. is shown. **(B)** Sphingomyelin levels in uninfected or LGTV-infected tick cells **(B)** or mock or GW4869 (1 μM)-treated, LGTV-infected tick cells **(D)** at 24 h and 72 h p.i. are shown. White bars represent uninfected (UI) and black bars denote LGTV-infected (I, MOI 1) groups. Mock represents group treated with vehicle DMSO. Both mock and GW4869 inhibitor-treated groups were infected with LGTV (MOI 1) for indicated time points. *Is*SMase activity and SM lipid levels were measured in milliunits/ml. *P*-value determined by Student's two-tailed *t*-test is shown. The asterisk indicates significance, and * or ** denotes a *P*-value of less than 0.05 or 0.01, respectively.

Since LGTV reduced *Is*SMase expression, we investigated whether the increased replication of LGTV (at time points of 24 and 72 h p.i., with MOI 1) also affects the *Is*SMase enzymatic activity and its function. We performed *Is*SMase activity assay in whole-cell lysates and found that LGTV significantly (*P* < 0.05) reduced *Is*SMase activity at an early time point (24 h p.i.) of infection (Figure 6A). However, no difference in the *Is*SMase activity was observed at a later time point (72 h p.i.) of LGTV infection (Figure 6A). Reduced activity of *Is*SMase upon LGTV infection suggested accumulating lipid metabolism pathway. Next, we determined the SM levels in whole tick cell lysates at 24 and 72 h post-LGTV infection. Quantification assay showed significantly (*P* < 0.05) increased SM levels upon LGTV infection at both time points of 24 and 72 h p.i., in comparison to the respective uninfected controls (Figure 6B). These data suggest that upon LGTV infection of tick cells, the reduced *Is*SMase expression and enzymatic activity/function perhaps lead to accumulation of SM lipid levels upon LGTV infection in ticks.

### GW4869 Treatment Restored *Is*SMase Activity by Suppressing LGTV-Induced SM Lipid Levels

Since GW4869 inhibitor treatment (at 1 μM) significantly affected the LGTV loads and restored the *Is*SMase transcript levels, next we analyzed the *Is*SMase enzymatic activity and SM levels. Tick cells treated with GW4869 inhibitor (1 μM for 4 h) followed by LGTV infection (MOI 1; at two different time points of 24 and 72 h p.i.) were analyzed for *Is*SMase activity and SM levels. We found that GW4869 inhibitor treatment restored the reduced *Is*SMase activity (Figure 6C). *Is*SMase activity was significantly (*P* < 0.05) increased upon GW4869 treatment and in a specific and early tested time point (24 h p.i.) of LGTV infection, in comparison to the mock control (Figure 6C). No further increase was observed at 72 h post LGTV infection in comparison to the mock control suggesting a complete restoration of *Is*SMase activity at an early time point (of 24 h p.i.,) (Figure 6C). Furthermore, GW4869 treatment significantly (*P* < 0.05) inhibited LGTV-induced SM lipid buildup at both 24 and 72 h p.i. in comparison to the respective mock LGTV-infected control groups (Figure 6D). These data indicate that GW4869 treatment inhibited LGTV loads resulting in increased *Is*SMase expression and activity that subsequently lead to reduction in SM lipid levels. Overall, these results suggest that a tick-borne flavivirus suppresses *Is*SMase expression and its activity to induce SM lipid levels that perhaps facilitate LGTV replication, packaging of viral RNA genomes and proteins, and budding of these virally activated exosomes.

## Discussion

Understanding the persistence, established colonization, and survival of pathogens for an extended period of time in medically important vector has been a topic of interest for several decades. The molecular mechanisms supporting the survival strategies have not been clearly understood for many of the vector-borne pathogens. Our previous study showed in detail how a tick-borne bacterium *A. phagocytophilum* induces the phosphorylation of fundamental molecule actin in order to selectively regulate the gene transcription of a salivary gland protein Salp16 to survive in *I. scapularis* ticks (Sultana et al., 2010). Proteins do phosphorylate and de-phosphorylate in a feedback mechanism, and this bacterium-induced phosphorylation of actin was an extended event that was observed for a lifelong time in ticks (Sultana et al., 2010). Our other important study showed that *A. phagocytophilum* induces anti-freeze glycoprotein (IAFGP) in *I. scapularis* ticks and establishes a beneficial/symbiotic relationship with its vector to survive in the cold (Neelakanta et al., 2010). Several other studies have also implicated molecules in regulating pathogen survival and transmission from the vector (Nuttall et al., 2000; Chambers and Diamond, 2003; Nuttall and Labuda, 2004; Piesman and Eisen, 2008; Kuhn et al., 2015; de la Fuente, 2018; Kim, 2019). An important study from Dr. Wikel's group identified a novel *Is*SMase, a gene with high homology to the *Loxosceles* venomous spider's SMase D protein in *I. scapularis* tick saliva (Alarcon-Chaidez et al., 2009). *Is*SMase regulated the expression and programming of IL-4, and a freeze-thaw stable structure within this molecule was proposed to bind a Toll-like receptor (TLR) or other receptor on antigen-presenting cells (like dendritic cells or monocytes) or innate immune cells that perhaps switches ON the Th2 differentiation (Alarcon-Chaidez et al., 2009).

In the current study, we determined the role of *Is*SMase in tick-borne flaviviral infection. It has been shown that the spider venom enzyme SMase D is in a gene family with multiple members that had variations in functional specificities and activities of this protein (Truett, 1993; Binford et al., 2005, 2009; Murakami et al., 2005; Fry et al., 2009; Pedroso et al., 2015). Several isoforms of SMase D were identified and characterized in different groups of spiders. Other than spiders, SMase D orthologs have also been identified in bacteria, fungi, and ticks (Binford et al., 2005; Dias-Lopes et al., 2013). Alignment of *Is*SMase (Q202J4), a novel sphingomyelinase-like enzyme (also known as Dermonectrotic toxin SPH; precursor), with SMase D from *Loxosceles* spp. spiders, suggested this tick molecule to be a venomous protein ortholog (Alarcon-Chaidez et al., 2009). Our detailed bioinformatics and comparative analyses further confirmed that *Is*SMase is a spider, SMase D venomous protein ortholog. Several isoforms of SMase D were identified and structurally classified into two groups. Class I SMase D proteins contained a single disulfide bridge and variable loops, whereas Class II proteins presented an additional intra-chain disulfide bridge that connected a flexible loop with a catalytic loop (Binford et al., 2009; Zobel-Thropp et al., 2010; Dias-Lopes et al., 2013; Pedroso et al., 2015). Toxic potential was, however, variable between the two classes, and the class II enzymes were less toxic than the Class I proteins (Binford et al., 2009; Zobel-Thropp et al., 2010; Dias-Lopes et al., 2013). Their phylogenetic data revealed that the Class I SMase D proteins are recent in their evolution driven by natural selection with an increased toxic variation and their origin from a single ancestor (Binford et al., 2009; Zobel-Thropp et al., 2010; Dias-Lopes et al., 2013). The protein structure has been solved for a member of this gene family (PDB 1XX1). This enzyme has a (α/ß)_8_ barrel structure with active sites that are dependent on binding of a Mg^2+^ ion for catalysis (Binford et al., 2009; Zobel-Thropp et al., 2010). ClustalW alignment of *I. scapularis* SMase D amino acid sequence showed higher identity with *R. pulchellus* or *A. maculatum* ticks. However, the phylogenetic analysis revealed that *Is*SMase formed a clade with spider venom protein orthologs from *S. patagonicus, H. lepturus*, and *L. similis*. The protein feature prediction analysis disclosed that *Is*SMase is a multifunctional protein with several active sites or motifs. The presence of several phospho-sites suggests that the process of phosphorylation perhaps regulates *IsSMase*.

**Figure 7:**
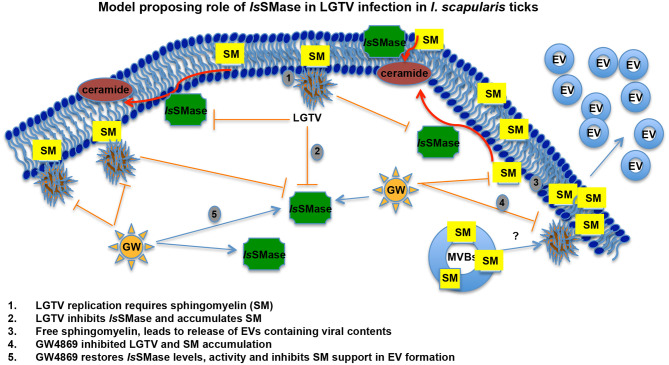
LGTV modulates sphingomyelinase and sphingomyelin lipids for its replication and survival in tick cells. Schematic representation with a proposed model on how LGTV plays a role in the suppression of sphingomyelinase levels and its activity to induce SM lipid levels for its own benefit in membrane association and viral replication processes. Treatment with GW4869 inhibitor reduced LGTV loads to restore the sphingomyelinase levels and activity, thereby inhibiting the buildup of SM lipids. We hypothesize that accumulated amounts of SM lipids not only support membrane-associated viral replication but also aid in the production and release of exosomes/EVs that mediate viral exit and dissemination. SM indicates sphingomyelin, EVs represents extracellular vesicles, and MVBs denotes multi-vesicular bodies.

The role of *Is*SMase in viral infections and pathogen transmission has not been studied. We have recently shown that medically important arthropods, such as ticks and mosquitoes, secrete extracellular vesicles including exosomes that mediate transmission of flavivirus RNA and proteins to vertebrate cells (Zhou et al., 2018). Our previous work showed that a tick-borne model pathogen LGTV readily infected *I. scapularis* (ISE6) tick cells, with increased viremia at 72 h p.i. and disseminated both positive and negative strands of LGTV RNA and viral proteins via secured exosomes (Zhou et al., 2018). Tick cell-derived exosomes enriched with exosomal marker HSP70 showed higher packaging of viral RNA and proteins. We noted induction in the number of exosome production and release from LGTV-infected tick cells and proposed this observation could result in enhanced viral dissemination (Zhou et al., 2018). Our previous study recommended analyzing the importance of neutral sphingomyelinase(s) in *I. scapularis* ticks upon LGTV infection. Our finding that LGTV dramatically reduced *Is*SMase levels in LGTV-infected unfed/fed ticks and in tick cells suggests that decrease in sphingomyelinase(s) leads to buildup of SM lipids that in turn may facilitate higher budding and release of exosomes. Furthermore, reversibility seen in restoring the expression of *Is*SMase upon GW4869 treatment suggested an involvement of this molecule in inhibiting LGTV replication and transmission via blocking/inhibition of exosome release and dissemination. We hypothesize that the venomous properties of *Is*SMase perhaps interfere with the viral replication by participating in tick anti-viral pathways. Upregulation of *Is*SMase expression and enzymatic activity upon GW4869 treatment perhaps suggests a negative role for this enzyme in exosome biogenesis. Combing of *I. scapularis* genome showed the presence of several other sphingomyelinases that are currently addressed in tick-LGTV interactions and exosome-mediated transmission of flaviviruses. In all animals, plants, and fungi, and in some of the prokaryotes and viruses, sphingolipids are the ubiquitous constituents of all the membranes, which include plasma membranes and membrane-bound organelles (Raposo and Stoorvogel, 2013; Schneider-Schaulies and Schneider-Schaulies, 2015; Bezgovsek et al., 2018; Shanbhogue and Hannun, 2018). In order to convert SM lipids into phosphocholine and ceramide, SMases have to be active and functional to hydrolyze this process (Clarke et al., 2006; Bartke and Hannun, 2009). SM consisting of phosphocholine and ceramide or a phospho-ethanolamine head group is a type of sphingolipid found in all animal membranes (Hannun and Obeid, 2018; Shanbhogue and Hannun, 2018). It represents 85% of all sphingolipids in human and accounts to 10–20 mol % of the plasma membrane lipids (van Meer and Lisman, 2002; Futerman, 2006; Bartke and Hannun, 2009). SM plays a critical role in signal transduction (van Meer and Lisman, 2002; Futerman, 2006; Bartke and Hannun, 2009). Sphingomyelinases are enzymes that hydrolyze SM to release phosphocholine into the aqueous environment and ceramide that diffuses through the plasma membrane (Clarke et al., 2006; Bartke and Hannun, 2009). Furthermore, there are several reports that are in positive notion that host lipids facilitate the genome replication of positive-strand RNA viruses (Apte-Sengupta et al., 2014; Vijayan and Hahm, 2014; Schneider-Schaulies and Schneider-Schaulies, 2015; Bezgovsek et al., 2018; Hannun and Obeid, 2018; Zhang et al., 2019). SM levels increased West Nile Virus (WNV) infection both *in vivo* and *in vitro* and have been suited as an antiviral target against WNV pathogenesis (Martin-Acebes et al., 2016). Dynamic remodeling of lipids, shown by alteration of the biochemical landscape of the mosquito midgut during dengue virus infection and replication suggested sphingolipids as “choke points” for targeting blocking of virus transmission (Chotiwan et al., 2018). Almost all well-studied positive-strand RNA viruses remodel and reorganize the membrane and lipid metabolism pathways through the coordinated virus-host interactions to create right niche or a suitable microenvironment for their survival and replication (Futerman, 2006; Zhang et al., 2013, 2019; Apte-Sengupta et al., 2014; Martin-Acebes et al., 2016; Chotiwan et al., 2018). Membrane components such as sphingolipids have been shown to participate in all steps of virus life cycles including membrane attachments and fusion, intracellular transport, replication, protein sorting, and budding/exogenesis of viral particles and virions (Futerman, 2006; Bartke and Hannun, 2009; Raposo and Stoorvogel, 2013; Schneider-Schaulies and Schneider-Schaulies, 2015; Hannun and Obeid, 2018). Influenza A virus (IAV) has been shown to activate the sphingosine kinase 1 and the transcription factor NF-kB, to manipulate the cellular signaling and the sphingosine metabolism as a hallmark for viral genome replication (Vijayan and Hahm, 2014; Soudani et al., 2019). In addition, human immunodeficiency virus (HIV) interacts directly with glycosphingolipids, and similar to HIV, hepatitis C virus (HCV) uses the lipid components for viral assembly and budding during viral infection and release (Hirata et al., 2012; Schneider-Schaulies and Schneider-Schaulies, 2015). Rhinoviruses stimulate the ceramide enrichment and endocytosis, whereas measles virus (MV) activates the sphingomyelinases (SMases) (Dreschers et al., 2007; Avota and Schneider-Schaulies, 2014; Schneider-Schaulies and Schneider-Schaulies, 2015). Interestingly, Sindbis viruses have been shown to replicate better in the absence of acid SMases (ASMases) (Jan et al., 2000; Ng and Griffin, 2006; Schneider-Schaulies and Schneider-Schaulies, 2015). Our study suggests that LGTV inhibits the expression and activity of *Is*SMase, in order to induce the production and accumulation of SM lipids (Figure 7). However, treatment with GW4869 (at only 1 μM), inhibited viral-induced SM lipid production and restored the *Is*SMase loads and activity as a feedback loop, suggesting a new role for this venomous ortholog in inhibition of tick-borne viral replication (Figure 7). Our data also suggest a specific role for *Is*SMase during LGTV infection, as ticks infected with *B. burgdorferi* or *A. phagocytophilum* bacteria did not show any significant difference in expression of *Is*SMase in comparison to levels noted in uninfected ticks (Supplementary Figure 3). This specific viral-mediated inhibition of *Is*SMase not only indicates a negative role or function for this enzyme in exosome-mediated transmission of virus but also suggests a new role for this venomous protein ortholog *Is*SMase in tick defense and/or anti-viral mechanism(s).

## Data Availability Statement

All datasets generated for this study are included in the article/Supplementary Material.

## Ethics Statement

The animal study was reviewed and approved by Institutional Animal Care and Use Committee (IACUC; protocol # 18-011).

## Author Contributions

PR, SK, GN, and HS performed experiments, and discussed, analyzed, and interpreted the data in several settings. GN generated the 24 h fed *I. scapularis* uninfected and LGTV-infected ticks. PR and SK performed RNA extractions and QRT-PCR. SK and GN performed bioinformatics analysis. PR and HS performed the quantification assays. All authors read and edited the manuscript. HS collected all required materials and reagents, designed and coordinated the entire study, compiled and organized all the data, supervised overall investigations, and wrote the paper.

## Conflict of Interest

The authors declare that the research was conducted in the absence of any commercial or financial relationships that could be construed as a potential conflict of interest.

## Supplementary Material

Supplementary Figure 1 Amplification of *I. scapularis* sphingomyelinase-like gene fragment (*Is*SMase) from ticks and tick cells. PCR amplification of *Is*SMase gene fragment from *I. scapularis* unfed or post-fed nymphal ticks or uninfected ISE6 tick cell line cDNA is shown. Similar size fragments were amplified from all three tested groups, and band of approximately 194 bp was detected on 1% agarose gel. Marker indicates size of the product amplified and NTC denotes no template control. M represents DNA ladder. Arrows indicate the 200 bp bands on ladder lane.

Supplementary Figure 2 Exosomes derived from tick cells had low or undetectable levels of *Is*SMase. QRT-PCR analysis showing detectable LGTV loads **(A)**, HSP70 transcript levels **(B)** and low or undetectable levels of *Is*SMase transcripts **(C)** in tick cell-derived exosomes that are uninfected (UI) or LGTV-infected (I) at day 1 and day 3 post infection (p.i.). Each circle, triangle, inverted triangle, or square represent sample generated from one culture well and analyzed in multiple replicate. Open circles indicate uninfected (UI) and closed circles denotes LGTV-infected group. LGTV loads, *hsp70* or *Is*SMase mRNA levels were normalized to tick beta-actin levels. *P*-value determined by Student's two-tail *t*-test is shown. The asterisk ^*^ indicates significance, and denotes a *P*-value of less than 0.05.

Supplementary Figure 3 *Is*SMase expression is unaffected in presence of other pathogens. QRT-PCR analysis showing levels of *Is*SMase transcripts in unfed *I. scapularis* nymphs infected with either extracellular bacterium *B. burgdorferi* or intracellular bacterium *A. phagocytophilum*. Open circles indicate uninfected (UI) and closed circles denote infected (I) groups. *Is*SMase mRNA levels were normalized to tick beta-actin levels. No significant differences were noted between UI and I groups.
