# Supplementary figures and images for: Tick-Borne Flavivirus Inhibits Sphingomyelinase (IsSMase), a Venomous Spider Ortholog to Increase Sphingomyelin Lipid Levels for Its Survival in Ixodes scapularis Ticks
Source: Front Cell Infect Microbiol. 2020 Jun 12;10:244. doi: 10.3389/fcimb.2020.00244 (PMC7325911; doi:10.3389/fcimb.2020.00244)

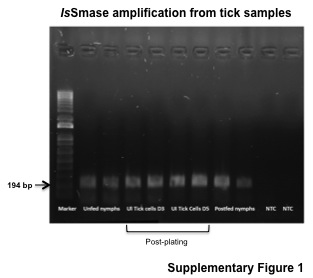

Supplement: Supplementary Figure 1 — Amplification of I. scapularis sphingomyelinase-like gene fragment (IsSMase) from ticks and tick cells. PCR amplification of IsSMase gene fragment from I. scapularis unfed or post-fed nymphal ticks or uninfected ISE6 tick cell line cDNA is shown. Similar size fragments were amplified from all three tested groups, and band of approximately 194 bp was detected on 1% agarose gel. Marker indicates size of the product amplified and NTC denotes no template control. M represents DNA ladder. Arrows indicate the 200 bp bands on ladder lane. [file Image_1.JPEG]

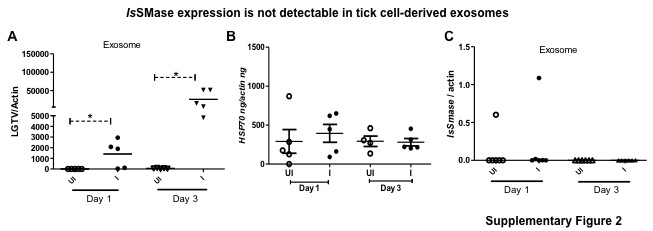

Supplement: Supplementary Figure 2 — Exosomes derived from tick cells had low or undetectable levels of IsSMase. QRT-PCR analysis showing detectable LGTV loads (A), HSP70 transcript levels (B) and low or undetectable levels of IsSMase transcripts (C) in tick cell-derived exosomes that are uninfected (UI) or LGTV-infected (I) at day 1 and day 3 post infection (p.i.). Each circle, triangle, inverted triangle, or square represent sample generated from one culture well and analyzed in multiple replicate. Open circles indicate uninfected (UI) and closed circles denotes LGTV-infected group. LGTV loads, hsp70 or IsSMase mRNA levels were normalized to tick beta-actin levels. P-value determined by Student's two-tail t-test is shown. The asterisk * indicates significance, and denotes a P-value of less than 0.05. [file Image_2.JPEG]

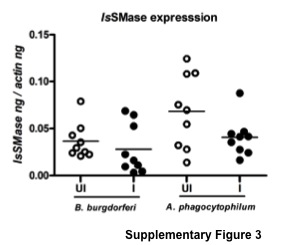

Supplement: Supplementary Figure 3 — IsSMase expression is unaffected in presence of other pathogens. QRT-PCR analysis showing levels of IsSMase transcripts in unfed I. scapularis nymphs infected with either extracellular bacterium B. burgdorferi or intracellular bacterium A. phagocytophilum. Open circles indicate uninfected (UI) and closed circles denote infected (I) groups. IsSMase mRNA levels were normalized to tick beta-actin levels. No significant differences were noted between UI and I groups. [file Image_3.JPEG]
